# New faster CHARMM molecular dynamics engine

**DOI:** 10.1002/jcc.23501

**Published:** 2013-12-02

**Authors:** Antti-Pekka Hynninen, Michael F Crowley

**Affiliations:** [b] Biosciences Center, National Renewable Energy LaboratoryGolden, Colorado, 80401

**Keywords:** CHARMM, molecular dynamics, parallel programming, domain decomposition

## Abstract

We introduce a new faster molecular dynamics (MD) engine into the CHARMM software package. The new MD engine is faster both in serial (i.e., single CPU core) and parallel execution. Serial performance is approximately two times higher than in the previous version of CHARMM. The newly programmed parallelization method allows the MD engine to parallelize up to hundreds of CPU cores.

## Introduction

CHARMM is a molecular dynamics (MD) software package with a wide range of capabilities beyond standard MD simulations.^1^,^2^ The capabilities include a powerful analysis tools, replica exchange MD, QM/MM, many advanced sampling methods, multiple implicit solvent methods, complex reaction coordinate restraints, path sampling, Monte Carlo sampling, string methods, and many advanced analysis tools. Although CHARMM is very useful for simulating practically any MD system, during the last few years its performance has been lagging behind other popular MD software packages such as GROMACS,^3^ NAMD,^4^ LAMPPS,^5^ and Amber^6^ in which developers have updated their algorithms and programming to reflect the invention of more powerful parallelization algorithms^7^,^8^ and changes in computer hardware.^1^

Our goal was to implement a modern parallel MD engine in CHARMM that performs at the same level as GROMACS, NAMD, LAMPPS, and Amber. The improvements to the performance in CHARMM are focused entirely on standard explicit-solvent, periodic systems using particle-mesh Ewald (PME) method^9^ for long-range electrostatics. The majority of the work is in the determination of nonbonded interactions and, in the PME method, is split into two uncoupled calculations: real space and reciprocal space contributions. The real-space contribution includes all the dispersion, or van der Waals (vdW), interaction and the real-space sum of Coulomb interactions. Each of the contributions has different challenges to improving performance and will be described separately. The real-space nonbond calculation in traditional MD programs usually is facilitated by restricting calculation of energies and forces between atoms that are separated by less than a cutoff distance. The real-space calculation is further enhanced by constructing a list of atom pairs within the cutoff, a list that is valid for some finite number of dynamics steps. The reciprocal calculation in the PME method comprises mainly a filling of a regular grid in the unit cell and a three-dimensional (3D) Fast Fourier Transform (FFT) and a sum in the reciprocal space. The spacing of the grid points determines the shape of the switching function in the electrostatic real-space calculation and can be adjusted so that the real-space Coulombic calculation vanishes at the same cutoff as the dispersion calculation. In this way, the same nonbonded pair list can be used for both calculations.

We present the improvements and describe the algorithms for performance on the single processor (serial) first and parallel performance second in the following sections. In the results section, we present benchmarks of the performance.

## Serial Performance Improvements

Table 1 shows timings for CHARMM MD using the old and new MD engines, and the speedup for a system of 23,558 atoms that was run for 1000 steps on a single CPU core. As can be seen from the last row of Table 1, the new CHARMM MD engine is, in total, two times faster than the old CHARMM MD engine. In the next subsections, we will go through the optimizations that gave rise to the speedups tabulated in Table 1.

**Table 1:** Timing data for CHARMM serial performance.

Routine/Timing	Old CHARMM (*s*)	New CHARMM (*s*)	Speedup
Nonbonded list builder	93.2	21.4	4.4
Direct nonbonded force	323.6	174.1	1.9
Reciprocal nonbonded force	67.2	37.3	1.8
Build charge grid	17.1	9.3	1.8
Scalar sum	15.0	5.5	2.7
Gradient sum	14.30	9.0	1.6
FFT	20.40	13.3	1.5
Total nonbonded force	399.8	214.3	1.9
Total time	503.9	252.2	2.0

The simulated system is DHFR (23,558 atoms), which was simulated for 1,000 steps on a single core of a 3GHz Quad-core Intel Xeon X5472 CPU.

### Direct part of the nonbonded force calculation

As we can easily confirm from Table 1, the direct part of the nonbonded force calculation takes approximately 60–70% of the total simulation CPU time. This part is by far the most important optimization target of the MD engine. We used three approaches to improve the performance in the direct part of the nonbonded force calculation on a modern CPU: we reduced code branching in the inner loop, wrote the inner loop using intrinsic streaming Single-Instruction-Multiple-Data (SIMD) Extension (SSE) operations and used lookup tables to evaluate the Coulomb and Lennard–Jones potentials.

To reduce branching, we wrote separate inner loops for solute–solute, solute–solvent, and solvent–solvent interactions. The split reduces branching, removes the need to check for 1–4 exclusions, reduces the size of the neighbor list by allowing us to put each solvent molecule as a single entry to the list, and allows us to do extra optimizations for loops with solvent molecules as described below.

Intrinsic SSE commands allow the programmer to take full advantage of the SIMD vector operations and hence maximize code performance. Intrinsic SSE operations are CPU hardware-dependent operations that very closely match the Assembler SSE operations found in the CPU. Although intrinsic commands make code tedious to write and harder to transfer to new hardware architectures, intrinsic C is more transferable and easier to write and than Assembler and yet the performance can be expected to be similar to pure Assembler code.

New nonbonded calculation inner loops were created from the original single inner loop to remove conditionals. Each loop is accessed with a separate nonbonded pair list as described later. In total, we have five versions of the inner loops:

1. Solute–solute with vdW and Coulomb interactions
2. Solute–solute with only vdW interactions
3. Solute–solvent with vdW and Coulomb interactions
4. Solute–solvent with only vdW interactions
5. Solvent–solvent with vdW and Coulomb interactions.

Note that inner loops 1 and 2 are different in the way that inner loop 2 calculates only the vdW interactions and no Coulomb interactions. Therefore, inner loop 2 is used to calculate interactions between solute atoms where at least one atom is without charge. Similar distinction applies to inner loops 3 and 4. In a typical simulation with solute and water as solvent, we create seven different nonbonded lists:

1. Solute–solute with vdW and Coulomb interactions
2. Solute–solute with 1–4 vdW and Coulomb interactions
3. Solute–solute with vdW interactions
4. Solute–solute with 1–4 vdW interactions
5. Solute–solvent with vdW and Coulomb interactions
6. Solute–solvent with vdW interactions
7. Solvent–solvent with vdW and Coulomb interactions.

Atom pair interactions are divided into these seven cases according to their atom type (solute vs. solvent), charge (zero charge vs. nonzero charge), and 1–4 interaction parameter. For example, solute-solute atom pair where both atoms have nonzero charge and no 1–4 interaction will go to list 1. If one of the solutes has zero charge, the pair would go to list 3. Solute-solvent pairs go to list 5 or 6 depending on the solute atom charge: if solute atom has zero charge, the pair goes to list 6 and if solute atom has nonzero charge, the pair goes to list 5.

The solvent-solvent inner loop 5 was optimized by loading the vdW parameters and the charges of solvent into variables at the beginning of the loop, therefore, removing the need for a table lookup of these parameters in the inner loop. The current version of the nonbonded force calculation routine only supports three-point water models.

The calculations of the vdW and Coulomb interactions are done using a spline interpolation on a precalculated lookup table. We split the vdW interaction into its attractive (*r*^6^) and repulsive (*r*^12^) components and perform the interpolation on each of them. This reduces the need for multiple lookup tables. Because each spline lookup entry consists of 4 values, the total size of a spline lookup entry is 12 values: 4 for attractive vdW, 4 for repulsive vdW, and 4 for Coulomb. For interaction types where the Coulomb interaction is not present, the lookup entry consists of 8 values.

There are essentially two fast methods to calculate Ewald and vdW interactions between a pair of atoms. Both methods use a lookup table because the Ewald Coulomb interaction involves the error function, which is slow to evaluate analytically. Method 1 avoids the calculation of square root and instead uses a lookup table that is indexed with the squared distance *r*^2^ to calculate the force. In case the value of energy is needed, a second lookup table is used. Method 2 calculates the square root and uses the distance *r* on a lookup table to calculate the energy. Force is obtained as a derivative of the energy. Note that to calculate force in method 2, the lookup table has to be differentiable.

We tested both methods and found that they had similar performance when we did not calculate energy in method 1 and used a hand written inverse square-root algorithm (see below) in method 2. The energy calculation slowed method 1 down, and the standard square-root operation slowed method 2. In Amber PMEMD and in the CHARMM “LOOKUP” code, method 1 is implemented such that potential energy is only calculated when needed. This happens rather infrequently, for example, when the state of the simulation is output on screen or written in a file.

Method 1 has the advantage that it can be efficiently implemented using a high-level programming language such as C or Fortran without resorting to intrinsic or Assembler programming. Method 2 has the advantage of giving both the energy and the force every time step without keeping track of when the energy evaluation is needed. Also, in method 2 a single inner loop can be used all the time, there is no need to write a separate one for the energy evaluation as in method 1. Because CHARMM assumes that the energy values are always up-to-date during a MD run, we chose to use method 2. Table 1 shows that the new direct nonbonded force calculation method is about 1.9 times faster than the previous version.

The hand-written inverse square-root algorithm consists of an approximate inverse square-root operation (RSQRTPS Assembler operation) and two iterations of the Newton–Raphson method. See the Ref. [10 for additional details on this method.

### Nonbonded neighbor list builder

A nonbonded neighbor list builder can be seen as the feeder routine of the nonbonded force calculation routine. It provides the nonbonded force calculation routine with a list of pairs of atoms that are within the cut-off radius and therefore, contribute to the nonbonded force. Because the structure of the nonbonded force calculation routine was changed, we needed to rewrite the nonbonded neighbor list builder. The new version of the neighbor list builder builds a separate list for each of the seven nonbonded calculations listed above.

We use a Verlet list where every atom *i* has a list of atoms *j* that are within the cut-off radius. The Verlet list is constructed using cell list method where the maximum size of the cells is set to half the neighbor list cut-off radius. The Verlet list is modified to include a pointer to a shift vector (*sx_i_, sy_i_, sz_i_*) for each atom *i*. Altogether there are 3 × 3 × 3 = 27 shift vectors, one for each possible minimum image shift. The shift vector gives the minimum image of atom *i* with respect to atoms *j*. One advantage of using the shift vector is that there is no need for calculating minimum images in the nonbonded force routine. Another advantage is that it allows for calculation of the virial pressure in the nonbonded force routine without much additional computational effort. Table 1 shows the new neighbor list builder is approximately 4.4 times faster than the previous version.

### Reciprocal part of the nonbonded force calculation

The reciprocal part of the PME calculation consists of four parts: placing atom charges on a grid, backward 3D FFT, scalar sum over the reciprocal grid to calculate energy and reciprocal force grid, forward 3D FFT of the grid, and gradient sum over the real-space grid to extract forces on atoms. We improved the performance of all four parts such that the total performance of the reciprocal nonbonded force calculation was improved by a factor of 1.8 (see Table 1).

We optimized the building of charge grid, scalar sum, and gradient sum routines by reorganizing loops to avoid branching in the innermost loops. Table 1 lists performance of these three operations showing the new routines are 1.8 times faster for charge grid build, 2.7 times faster for scalar sum, and 1.6 times faster for gradient sum.

The 3D FFT routine is implemented using the “column FFT” method where the FFT transforms are calculated along one single coordinate direction at a time in the following manner. Assume the charge grid consists of *G_x_* × *G_y_* × *G_z_* grid points. In the first step, we perform *G_y_* × *G_z_* real-to-complex FFT of length *G_x_* along the *x* direction. Because of the real-to-complex transformation, this results in a new grid of complex numbers with dimensions *(G_x_/*2 + 1*)* x *G_y_* × *G_z_*. We then transpose the grid from (*x, y, z*) to (*y, z, x*) order. The second FFT (complex-to-complex) of length *G_y_* is calculated along the *y* direction for every G_z_ × *(G_x_/*2 + 1*)* columns. We then transpose from (*y, z, x*) to (*z, x, y*) order. The third FFT of length *G_z_* is calculated along the *z* direction for every *(G_x_/*2 + 1*)* × *G_y_* columns. The backward FFT works the same way but in reverse, starting with FFT along the *z* direction and ending with the complex-to-real FFT along the *x* direction.

We optimized the 3D FFT routine by adding support for Fastest Fourier Transform in the West (FFTW)^11^ and Intel(C) MKL FFT libraries and we left the transpose routines as they were. In Table 1, the new CHARMM MD results use FFTW speeding up the entire 3D FFT by a factor 1.5.

## Improvements on Parallel Performance

**Figure 1:**
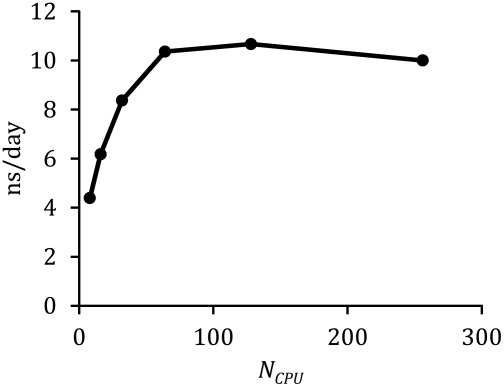
Parallel scaling of old CHARMM MD for 19,609 atoms.

In the previous version of CHARMM, the MD engine was parallelized by distributing the atoms evenly among the CPUs. That is, for *N* atoms and *N*_CPU_ CPUs, each CPU gets assigned *N*/*N*_CPU_ atoms. A CPU then calculates the pair interactions between its *N*/*N*_CPU_ atoms and all the atoms that are within the cut-off radius of these atoms. Because each CPU potentially needs the coordinates of all atoms, this method gives rise to all-to-all communications at each time step. In a typical simulation with about 20,000 atoms, the all-to-all communication starts to dominate the simulation time for *N*_CPU_ > 8, making it impossible to scale the simulation to many CPUs. Figure 1 shows the parallel scaling of the previous version of CHARMM for 19,609 atoms.

The new domain decomposition (domdec) version of CHARMM uses an internal group definition where each heavy atom along with any hydrogen atoms bonded to it form a single group. Note that with this definition, water solvent molecules are a single group. Because atoms are communicated among the nodes in groups of heavy atom plus its hydrogens, constraints on hydrogen bond lengths, angles, and dihedrals can be always performed within a single node without need for node-node communication. Currently, the code only supports SHAKE^12^ constraints between heavy atom and hydrogens.

### Domain decompostion parallel method

To improve parallel scaling of CHARMM, we implemented a domain decomposition method called “eighth-shell” where the atoms are assigned to CPUs according to their spatial position rather than their position in the atom array. The eighth-shell method was first introduced by Shaw and coworkers^7^ as one of the variants of what they call “zonal” methods. In the eighth-shell method, the simulation box is divided into *N_x_* × *N_y_* × *N_z_* subboxes, where *N_x_*, *N_y_*, and *N_z_* are the number of subboxes in *x*-, *y*-, and *z*-directions. Each CPU is assigned one subbox, called “home-box.” A CPU is responsible for the atom groups that are within its home-box.

**Figure 2:**
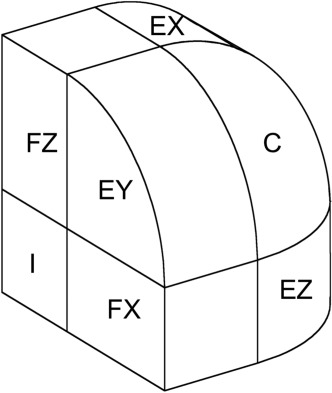
Import volume zones. Pair interactions are calculated between zones: I-I, I-FX, I-FY, I-FZ, I-EX, I-EY, I-EZ, I-C, FX-FY, FX-FZ, FX-EX, FY-FZ, FY-EY, and FZ-EZ.

To calculate the nonbonded interactions, a CPU needs the coordinates of atoms that are cut-off radius *R*_cut_ distance from the home box boundary. In 3D, this gives rise to a so-called “import volume” around the home box. Figure 2 illustrates the import volume, where the home box is marked with I and the import volume is divided into seven zones: FX, FY, FZ, EX, EY, EZ, and C. Note that zone FY is not visible in Figure 2.

To correctly calculate all the nonbonded interactions, all particles in the following zone pairs need to be calculated: I-I, I-FX, I-FY, I-FZ, I-EX, I-EY, I-EZ, I-C, FX-FY, FX-FZ, FX-EX, FY-FZ, FY-EY, and FZ-EZ.

The communication pattern between the nodes was setup in three steps:

Let the node home box be located at (*x, y, z*) where *x* = 1 … *M_x_*, *y* = 1 … *M_y_*, and *z* = 1 … *M_z_*. And *M_x_*, *M_y_*, and *M_z_*, are the number of nodes within the cut-off radius in each direction, for example, *M_x_* = ceiling (*R*_cut_/*D_x_*), where *D_x_ = L_x_/N_x_* is the *x* dimension of the subbox. In “Step Z,” the node at (*x, y, z*) sends the required coordinates to the nodes (*x, y, z* − 1) … (*x, y, z − M_z_*). At the same time, the node (*x, y, z*) receives coordinates from nodes (*x, y, z* + *1*) … (*x, y, z* + *M_z_*). In the next step, “Step Y,” the node sends the required coordinates to nodes (*x, y* − *1, z*) … (*x, y* − *M_y_, z*). Note that some of the coordinates that are sent in this step include coordinates that were received in Step Z. In the last step, “Step X,” the node sends the required coordinates to nodes (*x* − *1, y, z*) … (*x* − *M_x_, y, z*). Again, some of the coordinates that are sent in this step include coordinates that were received in Step Z and Step Y. In this method, every node sends and receives *M_z_* + *M_y_* + *M_x_* coordinate sets. The program then assigns the received coordinates to the appropriate zones.

### Reciprocal space parallel approach

**Figure 3:**
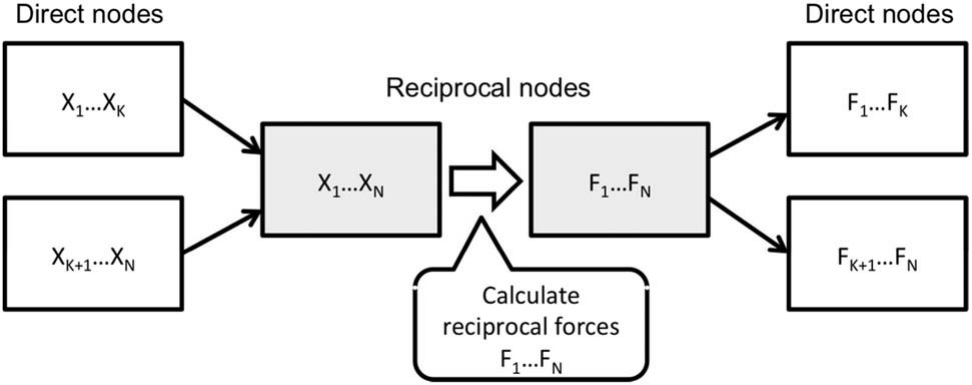
Direct-reciprocal communication pattern.

We split the direct space and reciprocal space PME calculation onto separate sets of nodes. The direct nodes run the regular MD engine code and do all the calculations apart from the reciprocal force calculation. At the beginning of the force calculation, the direct nodes send the coordinates to their reciprocal peer node. Note that each direct node has exactly one reciprocal peer node. After the communication, the direct nodes return and continue calculating bonded and nonbonded forces. The reciprocal nodes do an all-to-all communication on the coordinates and then perform the reciprocal nonbonded force calculation using the method described earlier. After the force calculation, the reciprocal nodes perform another all-to-all communication on the forces. In the last step, the reciprocal nodes send the forces to their direct peer nodes. Figure 3 illustrates this communication pattern for the case when there are two direct nodes and one reciprocal node.

### Load balancing

The major source of load imbalance during a simulation run is the different amount of work performed by the direct nodes in the nonbonded force calculation routine. The load difference arises somewhat from local density differences, and more importantly, from the fact that the force calculation for the solvent molecules executes faster than that for the solute molecules. At the communication step, nodes that have less work have to wait for nodes that have more work resulting in wasted CPU time.

We implemented dynamic load balancing for the direct nodes by changing the size of the home boxes during a simulation in a manner similar to the method used in GROMACS^3^. In this method, nodes with high load will shrink, whereas nodes work low load will expand, thereby balancing out the load and resulting in less wait time.

**Figure 4:**
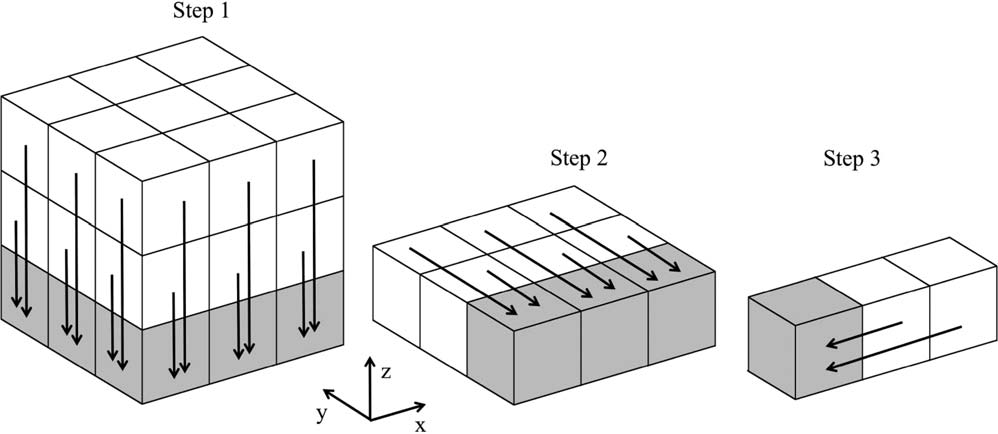
Communication pattern in dynamic load balancing. Receiving nodes are colored gray and the arrows denote direction of data transfer.

Dynamic load balancing is done at each neighbor list update and the load for each node is defined as the total time spent in the nonbonded force calculation routine between neighbor list updates. We measure the time using the standard wall clock timer. The communication of the timing data is illustrated in Figure 4. In the first step, *z* = 1 nodes receive timings from nodes *z* > 1 to variable timeZ(1:*N_z_*) [where timeZ(1) is the timing of node *z* = 1 itself]. In the second step, nodes at *z* = 1 and *y* = 1 receive Sum(timeZ(1:*N_z_*)) from the nodes *z* = 1, *y* > 1 to variable timeY(1:*N_y_*). In the third step, node at *z* = 1, *y* = 1, and *x* = 1 receives Sum(timeY(1:*N_y_*)) from the nodes *z* = 1, *y* = 1, and *x* > 1 to variable timeX(1:*N_x_*).

**Figure 5:**
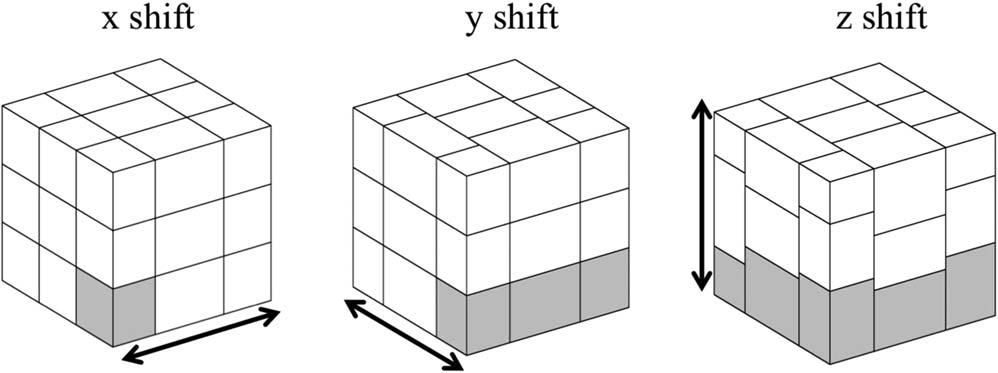
Shifting of *x*, *y*, and *z* boundaries in the dynamic load balancing algorithm. The arrows denote the direction in which the shift is done and the gray color denotes the nodes that make the shifting decisions.

Figure 5 illustrates the node boundary shifting procedure. In the first step called “x shift,” node at *z* = 1, *y* = 1, and *x* = 1 uses the timing data timeX(1:*N_x_*) to shift the *x* boundaries and sends the new shifted boundaries to the nodes at *z* = 1, *y* = 1, and *x* > 1. In the second step, nodes with *z* = 1, *y* = 1, *x* >= 1 use the data timeY(1:*N_y_*) to shift the *y* boundaries and send the results to nodes with *y* > 1. In the last step, nodes with *z* = 1, *y* >= 1, *x* >= 1 use the data timeZ(1:*N_z_*) to shift the z boundaries and send the results to nodes with *z* > 1.

**Figure 6:**
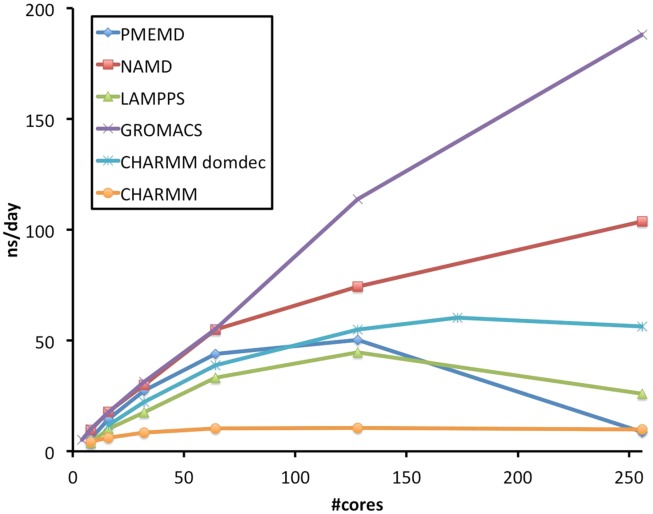
Benchmarks for Crambin with 19,609 atoms.

**Figure 7:**
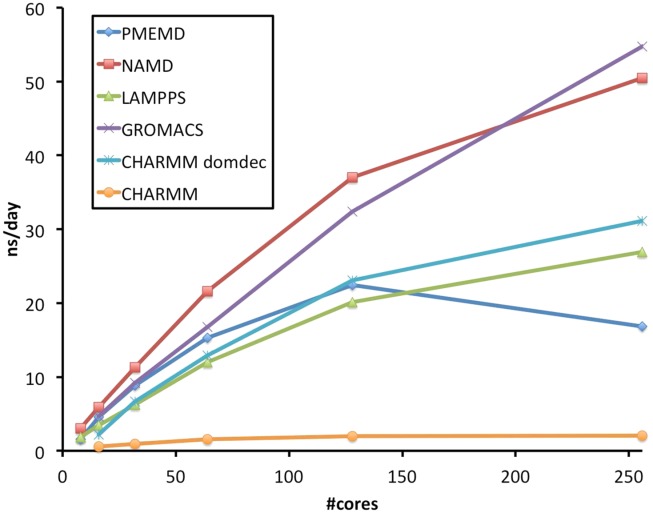
Benchmarks for GlnBP with 61,157 atoms.

For node *i* = 1, …, *N_x_* load imbalance in *x* direction,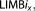 is defined as,
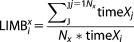


That is, load imbalance for node *i* is defined as the average time divided by the time spent on node *i*. Nodes that spend more than average time in the direct nonbonded calculation have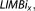 smaller than 1.0 indicating that the size of the subboxes belonging to these nodes should be reduced in the *x* direction. Load imbalances for *y* and *z* directions are defined similarly.

### Bonded terms for energy and forces

When assigning bonded interactions (bonds, angles, dihedrals) and SHAKE groups to a node, we must make sure that (a) the node has the coordinates and (b) only a single node is assigned to each interaction or SHAKE group. To simplify the terminology, we will from now on call groups of atoms involved in a bonded interaction or SHAKE groups as simply “bonded group.”

Both requirements are satisfied by taking the minimum of the *x*, *y*, and *z* coordinates of the bonded group and assigning the group to the node with the home box where this coordinate resides. Note that in this rule the minimum coordinate is the minimum of *x*, *y*, and *z* separately and therefore, is not necessarily the coordinate of any of the atoms in the group.

Dynamic load balancing causes a slight modification to this rule because it is possible to have the minimum assigned to a node that does not have all the coordinates. The algorithm to resolve this issue is rather tedious and will not be explained here.

### Constraints and restraints

Current version of DOMDEC supports the distance matrix constraints (CHARMM command DMCONS), umbrella sampling (CHARMM command RXNCOOR), and absolute harmonic constraints (CHARMM command CONS HARM ABS). In the absolute harmonic constraints method, an atom is constrained to a spatial location. Because only local atom coordinate information is needed in this method, it was easily implemented in DOMDEC. In contrast, in both distance matrix constraints method and umbrella sampling method, the spatial extent of the constraints are not limited but can span the entire simulation box. Therefore, these methods cannot be implemented locally on the direct PME nodes and we instead implemented them on the reciprocal PME nodes that have the entire atom coordinate set. In particular, we chose the root node of the reciprocal node set to be the “constraints” node.

## Usage

The new MD engine described in this article is activated by adding the keyword “DOMDEC” in the ENERGY command in CHARMM script. See the CHARMM documentation for details of how to turn on SHAKE and restraints. Without any options, DOMDEC will start the simulation with dynamic load balancing on and will determine the number of direct and reciprocal CPU cores. The full DOMDEC options are

NDIR NX NY NZ

DLB ON | OFF

As an example, “DOMDEC NDIR 2 2 2 DLB OFF” will start DOMDEC with eight (*N_x_* = 2, *N_y_* = 2, and *N_z_* = 2) direct nodes and with dynamic load balancing off. The rest of the nodes are used as reciprocal nodes. If the simulation was started with “mpirun − n 12,” there would be eight direct nodes and 12 − 8 = 4 reciprocal nodes. If the simulation was started with “mpirun − n 8,” then the direct nodes will also act as reciprocal nodes, that is, there will be no split into direct and reciprocal. Because the reciprocal nodes need to perform all-to-all communication, it is not advisable to use this strategy for a large number of MPI nodes.

The use of DOMDEC limits the kind of MD simulations that can be performed. Here is a partial list of limitations

1. Only supports orthorhombic/orthogonal simulations with perpendicular axes and full periodicity in all 3D. General triclinic unit cells (truncated octahedron, rhombic dodecahedron) and unconventional crystal symmetries such as fivefold symmetry, and symmetries with rotations/reflections are not yet supported.
2. Only supports three-point solvent models
3. Only supports the leapfrog (LEAP), Langevin (LANG), and constant pressure (CPT) integrators
4. Only supports PME electrostatics
5. Does not support Drude oscillator model or lonepairs.

We are currently implementing more CHARMM features in DOMDEC. Apart from the unconventional crystal symmetries (fivefold symmetry, symmetries with rotation/reflection, etc.), there is no restriction preventing support for most CHARMM features in DOMDEC. For example, TIP4P solvent model, Drude oscillators, lonepairs and support for other integrators can all be implemented in DOMDEC. Unconventional crystal symmetries are harder to implement because they do not fit well with our domain decomposition scheme. To implement these, one should use a different decomposition scheme or resort back to the traditional CHARMM way of splitting the atom list among the nodes.

## Results

### Validation

We validate the new MD engine by comparing the results with the standard CHARMM MD engine. For this purpose, we include five test cases for the DOMDEC command in the distributed CHARMM test suite: MD simulation run of DHFR (domdec.inp), distance matrix constaint (domdec_dmcons.inp), harmonic absolute constraint (domdec_cons_harm.inp), vdW nonbonded force (domdec_vdw.inp), and electrostatic nonbonded force (domdec_elec.inp). These tests make sure DOMDEC gives the same results as the standard CHARMM.

The MD simulation run test (domdec.inp) runs for 100 MD steps with and without DOMDEC and the resulting total energies are compared. The energy from the run with DOMDEC matches with the energy from the standard CHARMM up to four decimal places with relative error around 2 × 10^−9^. This and other tests we performed on the code give us confidence that the MD engine is working correctly.

One of the potential bugs in any domain decomposition code is the incorrect distribution of bonded interactions among the nodes. DOMDEC checks for this bug by making sure the total number of bonded interactions (bonds, angles, and dihedrals) as well as the total number of SHAKE constraints over all direct PME nodes are correct. This check is done at every MD step and it enabled us to find many bugs in the code.

### Benchmarks

Benchmarking was done on three solvated systems: Crambin with total of 19,609 atoms, GlnBP with 61,157 atoms, and hEGFR with 465,404 atoms. Details of these systems, developed at the STFC Daresbury Laboratory, can be found in the Ref. [13. We ran the Crambin and GlnBP systems for 50,000 steps and the hEGFR system for 10,000 steps. The size of the time step was 2 fs, and all simulations were performed in the constant pressure, constant temperature ensemble. Benchmarking was performed on an infiniband Linux cluster that has two Intel(R) Xeon(R) E5620 (2.40GHz) CPUs per node. In total, then each CPU node has eight cores.

**Figure 8:**
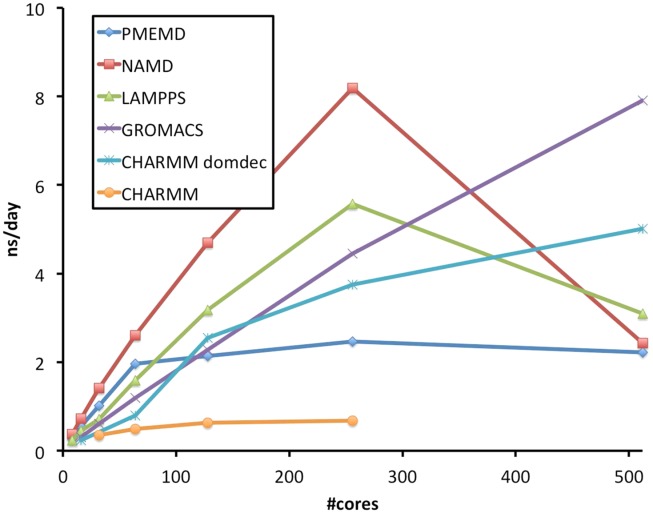
Benchmarks for hEGFR with 465,404 atoms. [Color figure can be viewed in the online issue, which is available at wileyonlinelibrary.com.]

The benchmarks were run using PMEMD Amber 11, NAMD version 2.9, GROMACS version 4.5.5, and CHARMM version c37b1 software. Figures 8 show the timings for Crambin in ns/day. Across the board, GROMACS and NAMD perform the best, whereas the performance of CHARMM with domdec is in the midrange, similar to LAMPPS and PMEMD. The performance of CHARMM without domdec is clearly the worst. Comparing Figures 8 shows that NAMD performs better than GROMACS for large systems.

## Conclusions

We have introduced modernized MD engine to CHARMM software package that performs at the level similar to other popular MD engines.

We are looking into tuning the CHARMM DOMDEC MD engine further as we believe it has potential for still better performance. The other features of CHARMM are being migrated into the DOMDEC MD code for future releases.

## Footnotes

† One of the most important recent changes in computer hardware is the introduction of SIMD (Single Instruction Multiple Data) vector instructions.
